# Impact of Antithrombotic Therapy on the Outcome of Patients Undergoing Laparoscopic Colorectal Cancer Surgery: A Systematic Literature Review

**DOI:** 10.7759/cureus.23390

**Published:** 2022-03-22

**Authors:** Takahisa Fujikawa, Ryo Takahashi

**Affiliations:** 1 Surgery, Kokura Memorial Hospital, Kitakyushu, JPN

**Keywords:** thromboembolic complication, bleeding complication, antithrombotic therapy, colorectal cancer surgery, laparoscopic surgery

## Abstract

In recent years, many operations have been performed as laparoscopic surgeries in the field of gastrointestinal surgery, but the effect of antithrombotic therapy (ATT) on hemorrhagic complications in patients who have undergone laparoscopic colorectal cancer surgery remains unknown. In addition, the efficacy and safety of pharmacotherapy for the prevention of venous thromboembolism (VTE) have not yet been concluded. The purpose of this systematic review study is to clarify the effect of ATT on hemorrhagic complications in patients undergoing laparoscopic colorectal cancer surgery.

Articles published between 2013 and 2020 were searched on Google Scholar and PubMed, and research regarding ATT and laparoscopic colorectal cancer surgery was included after a thorough examination of each study. Each study yielded information on the study's design, type of surgical procedures, antithrombotic medications used, and surgical outcomes (both thromboembolic and hemorrhagic consequences).

This systematic review comprised 20 published papers, including a total of 12,751 patients who received laparoscopic colorectal cancer surgery. Four studies on thrombosis prevention in VTE were randomized clinical trials, and the other 16 were cohort or case-control studies. For the effects of prolonged use of ATT on hemorrhagic complications, most studies demonstrated that laparoscopic colorectal cancer surgery with continued preoperative aspirin could be safely conducted without an increase in the frequency of bleeding complications. On the other hand, most included papers have shown that patients receiving VTE pharmacoprophylaxis may be at an increased risk of bleeding complications, but its effectiveness has not been statistically proven, especially in the Asian patient population.

Laparoscopic colorectal cancer surgery in patients on prolonged ATT can be safely conducted with no increase in the incidence of hemorrhagic or thrombotic complications. The efficacy and safety of VTE pharmacoprophylaxis in laparoscopic colorectal surgery is still at issue. It is necessary to establish available protocols or guidelines by validating reliable studies.

## Introduction and background

The three leading causes of death in the world are cancer, cerebrovascular disease, and heart disease. With the aging of society in recent years, the risks for patients suffering from cerebrovascular disease and cardiac disease to require non-cardiac surgery are expanding. Most of these patients receive antithrombotic therapy (ATT) to prevent thromboembolism, but patients undergoing ATT are at an increased risk of both thromboembolism and bleeding during the perioperative period. Therefore, strict antithrombotic drug management is required [1].

ATT is classified into two types: antiplatelet therapy and anticoagulant therapy. Antiplatelet agents are used for primary and secondary prevention of cerebrovascular and cardiovascular diseases, the mechanism of which is to prevent thrombosis by suppressing platelet aggregation [2,3]. Anticoagulants are primarily used for acute coronary syndrome, deep venous thrombosis, atrial fibrillation, post-cardiac prosthesis replacement, and venous thromboembolism (VTE) pharmacoprophylaxis, the mechanism of which is to prevent blood coagulation by inhibiting the native coagulation cascade [3]. Table 1 summarizes the types of antithrombotics, specific drugs, and duration of action. Antiplatelet agents include thienopyridines (e.g., clopidogrel, ticlopidine, or prasugrel), acetylsalicylic acid (aspirin), type III phosphodiesterase inhibitors (e.g., cilostazol), and other nonsteroidal anti-inflammatory drugs. Anticoagulants include unfractionated heparin, heparin derivatives (e.g., fondaparinux), low-molecular-weight heparin (e.g., dartepalin, enoxaparin), vitamin K antagonists (e.g., warfarin), and direct-acting oral anticoagulants (DOACs, also known as non-vitamin K antagonist oral anticoagulants). DOAC is further divided into factor Xa inhibitors (e.g., apixaban, rivaloxaban, edoxaban) and direct thrombin inhibitors (dabigatran).

**Table 1:** Types, specific agents, and acting duration of commonly used antithrombotic drugs. DOAC, direct-acting oral anticoagulant;  iv, intravenous; LMWH, low-molecular-weight heparin; NSAID, non-steroidal anti-inflammatory drug; PDE, phosphodiesterase; sc, subcutaneous.

Agent class	Type of agent	Specific drugs	Duration of action
Antiplatelet agent			
	Thienopyridines	Clopidogrel	5-7 d
		Prasugrel	5-7 d
		Ticlopidine	10-14 d
		Ticagrelor	5-7 d
	Type III PDE inhibitor	Cilostazol	2 d
	Acetylsalicylic acid	Aspirin	7-10 d
	Other NSAIDs	Ibuprofen, loxoprofen, diclofenac etc.	Varies
Anticoagulation agent			
	Unfractionated heparin	Heparin	1-2 h
	LMWH	Dalteparin (iv)	2-4 h
		Enoxaparin (sc)	6-12 h
		Nadroparin (sc)	6-12 h
	Vitamin K antagonist	Warfarin	5 d
	Factor Xa inhibitor (sc)	Fondaparinux (sc)	1-1.5 d
	DOAC: Direct thrombin inhibitor	Dabigatran	1-2 d
	DOAC: Factor Xa inhibitor	Rivaroxaban	1-2 d
		Apixaban	1-2 d
		Edoxaban	1-2 d

In recent years, many operations in the field of gastrointestinal surgery have been performed as laparoscopic surgeries. Benefits of laparoscopic surgery have been shown to include a decrease in postoperative complications, reduced postoperative pain, and a quick return to social life [4,5], although it is still under debate whether these benefits can minimize the risk of thromboembolism during laparoscopic surgery [2]. Laparoscopic surgery has been reported to significantly reduce intraoperative bleeding [4,5]. Maintaining a bleeding-free surgical field is very important in laparoscopic surgery, and with improvements in various surgical techniques and the introduction of new surgical devices such as soft coagulation systems or ultrasonic coagulating shears, various sorts of advanced laparoscopic surgery, including colorectal cancer resection, can currently be performed. On the other hand, optimal perioperative management of patients undergoing ATT for laparoscopic colorectal cancer surgery is still under discussion.

The aim of this systematic review is to clarify the effect of ATT on thromboembolism and bleeding complications (BCs) in laparoscopic colorectal cancer surgery.

## Review

Methods

English-written articles published between 2013 and 2020 were searched by Google Scholar and PubMed. The relevant keywords, including aspirin, clopidogrel, warfarin, anticoagulant, antiplatelet, hemorrhage, bleeding, laparoscopic surgery, and colorectal cancer surgery, were used in the search. We have selected research articles that were published in peer-reviewed journals. Randomized clinical trials, case-control studies, or prospective or retrospective cohort studies were all considered eligible; guidelines, review papers, and case series/reports were not.

After duplicates were removed, each study was carefully reviewed and papers were methodically discarded. Depending on the study design, the quality of each study was evaluated, and relevant papers were identified. Each study yielded information on the study's design, type of surgical procedures, antithrombotic medications used, and surgical outcomes (both thromboembolic and hemorrhagic consequences). Increased surgical blood loss (SBL) and postoperative BCs were the two types of hemorrhagic consequences.

Results

Features of the Included Studies

Research screening and collection were conducted from December 2020 to January 2021. In total, we analyzed 20 published articles, with a total of 12,751 patients undergoing laparoscopic colorectal cancer surgery. The eligible articles consisted of 11 studies regarding the management of patients with prolonged ATT (Table 2) [2,6-15] and nine studies regarding pharmacological thromboprophylaxis for VTE (Table 3) [16-24]. Four studies concerning thromboprophylaxis for VTE were randomized clinical trials, and the other 16 were cohort studies or case-control studies. Ten were observational cohort studies, all of which were retrospective in nature. Concerning the research on the management of patients with prolonged ATT, one study was a multicenter retrospective cohort analysis [8] and three were analyses using the propensity score matching method [6,9,11].

**Table 2:** Reported data concerning bleeding complications of laparoscopic colorectal surgery in patients with antithrombotic therapy. ACT, anticoagulation therapy; APT, antiplatelet therapy; ATT, antithrombotic therapy; BC, bleeding complication; CCS, case-control study; LAP, laparoscopic; mRCS, multicenter RCS; PSM, CCS with propensity-score matching; RCS, retrospective cohort study; SBL, surgical blood loss; TE, thromboembolism.

Author of each report	Year, type	Surgery type	Drug use and exposure	Bleeding events	TE, mortality
Takahashi [6]	2020, PSM	Laparoscopic colorectal cancer surgery	Patients not on continued APT (n=649, control) vs patients with continued aspirin (n=140); post-PSM: 105 vs 105 matched cases	BC 1.0% in control (P=0.317) vs 2.9% in continued aspirin; SBL was comparable (P=0.068)	TE 1.0% in control vs 0% in continued aspirin; mortality 1.9% vs 0% (P=0.155)
Ohya [8]	2020, mRCS	Laparoscopic colorectal cancer surgery	Patients not on continued APT (n=125, control) vs patients with continued aspirin (n=89)	BC 2.4% in control vs 4.5% in continued aspirin (P=0.453); SBL was comparable	TE 2.4% vs 0% (P=0.268); mortality 0.8% vs 1.1% (P=1.000)
Fujikawa [7]	2020, RCS	Major digestive surgery including laparoscopic colorectal surgery	Patients not on APT (n=2019, control) vs patients with discontinued APT (n=542) vs patients with continued aspirin (n=421)	BC 1.3% in control vs 3.5% in discontinued APT vs 3.8% in continued aspirin; BC rate comparable after adjusting (P>0.05)	TE 0.5% in continued aspirin or control vs 2.8% in discontinued APT (P<0.001); mortality 0.7%/0.6% vs 1.1% (P=0.340)
Taguchi [9]	2019, PSM	Laparoscopic colorectal surgery	Patients not on continued APT (n=427, control) vs patients with continued aspirin (n=36); post-PSM: 36 vs 36 matched cases	BC 2.8% in control vs 0% in continued aspirin (P=0.237); SBL was comparable (P=0.503)	No TE event in both groups; no mortality in both groups
Yoshimoto [10]	2019, RCS	Laparoscopic colorectal surgery	Patients not on APT (n=410, control) vs patients with discontinued APT (n=114) vs patients with continued aspirin (n=54)	BC 1.2% in control vs 0.9% in discontinued APT vs 1.9% in continued aspirin (P=0.864); SBL was comparable (P=0.304)	TE 0.5% vs 1.8% vs 0% (P=0.287); no mortality in whole cohort
Nozawa [11]	2018, PSM	Laparoscopic colon cancer surgery	Patients without ATT (n=618, control) vs patients with ATT (n=96); post-PSM: 93 vs 93 matched cases	BC 1.1% in control vs 2.2% in ATT (P>0.05); SBL was comparable	No TE event in both groups
Nozawa [12]	2019, CCS	Laparoscopic rectal cancer surgery	Patients without ATT (n=332, control) vs patients with ATT (n=32)	BC rate was comparable; SBL was comparable	TE rate was comparable
Shimoike [13]	2016, RCS	Colorectal cancer surgery including laparoscopic surgery	Patients without APT (n=343, control) vs patients with APT (n=148)	BC 0.9% in control vs 0.7% in APT (P=1.000)	TE 0% vs 0.7% (P=0.301); no mortality in both groups
Sulu [14]	2013, CCS	Colorectal surgery including laparoscopic surgery in those w/ACT	Patients undergoing open surgery (n=159, control) vs patients undergoing LAP surgery (n=102)	Postop hemoglobin levels higher in LAP; blood transfusion rate was comparable	VTE 24.5% in control vs 2.9% in LAP (P<0.001)
Ono [15]	2013, CCS	Laparoscopic colorectal cancer resection and laparoscopic cholecystectomy	Patients without aspirin (n=436, control) vs patients with continued aspirin (n=52)	SBL 17 mL in control vs 27 mL in continued aspirin (P=0.430)	No mortality in both groups
Fujikawa [2]	2013, RCS	Laparoscopic surgery including laparoscopic colorectal surgery	Patients not on APT (n=863, control) vs patients with discontinued APT (n=160) vs patients with continued aspirin (n=52)	BC 0.7% in control (P=0.987) vs 2.5% in discontinued APT vs 0% in continued aspirin; BC rate identical after adjusting	TE 0.2% vs 0.6% vs 0.5% (P=0.625); only one mortality in continued aspirin (1.9%)

**Table 3:** Reported data concerning the safety of thromboprophylaxis for venous thromboembolism during laparoscopic colorectal surgery. AOR, adjusted odds ratio; BC, postoperative bleeding complication; CR, clinically relevant; LAP, laparoscopic; LMWH, low-molecular-weight heparin; mRCS, multicenter retrospective cohort study; mRCT, multicenter randomized controlled trial; PE, pulmonary embolism; RCS, retrospective cohort study; TP, thromboprophylaxis; VTE, venous thromboembolism.

Author of each report	Year, type	Surgery type	Drug use and exposure	Bleeding events	TE, mortality
Kamachi [16]	2020, mRCT	Laparoscopic colorectal surgery and laparoscopic gastric surgery	Patients with TP (LMWH; enoxaparin, n=182) vs patients w/o TP (control, n=208)	BC 5.4% in TP (11/182); one patient with major BC in TP	VTE 3.3% in TP vs 4.8% in control (P=0.453); CR-VTE 0.5% vs 3.4% (P=0.050)
Nakagawa [17]	2020, mRCT	Laparoscopic colorectal cancer surgery	Patients with TP (LMWH; enoxaparin, n=61) vs patients w/o TP (control, n=60)	BC 1.8% in TP vs 0% in control (P>0.05)	VTE 12.3% vs 11.9% (P=1.00)
Hata [18]	2019, mRCT	Laparoscopic colorectal cancer surgery	Patients with TP (LMWH; enoxaparin or fondaparinux, n=145) vs patients w/o TP (control, n=157)	Overall BC 13.1% in TP vs 3.2% in control (P=0.002); major BC 1.4% vs 1.3% (P=0.936)	VTE 2.8% vs 5.1% (P=0.293); no symptomatic VTE in whole cohort
Pak [19]	2018, RCS	Colorectal cancer surgery including laparoscopic surgery	Patients with TP (LMWH; fondaparinux, n=62) vs patients w/o TP (control, n=484)	BC 11.3% in TP vs 4.5% in control (P=0.046); BC rate comparable after adjusting	(not mentioned)
Tokuhara [20]	2017, RCS	Laparoscopic colorectal cancer surgery	Patients with TP (LMWH; fondaparinux, n=128, single arm)	Overall BC 6.7% in TP; major BC 1.7% in TP	VTE 2.5% in TP (all is DVT); no PE in whole cohort
Yasui [21]	2017, mRCS	Colorectal cancer surgery including laparoscopic surgery in patients w/ LMWH (fondaparinux)	Patients receiving LAP surgery (n=419) vs patients receiving open surgery (control, n=200)	Overall BC 11.9% in LAP vs 7.0% in control (P=0.059); major BC 0.7% in LAP vs 1.0% in control (P=0.519)	No CR-VTE in whole cohort
Iannuzzi [22]	2016, mRCS	Colorectal cancer surgery including laparoscopic surgery	Patients with colorectal surgery (n=128,163)	(Not mentioned)	Post-discharge VTE 0.7% in all cohorts; reduced AOR in LAP surgery (AOR=0.80, P=0.010)
Monghadamyeghaneh [23]	2016, mRCS	Colorectal cancer surgery including laparoscopic surgery	Patients with colorectal surgery (n=219,477, from NSQIP database)	(Not mentioned)	Overall and post-discharge VTE 2.1% and 0.7%, respectively; increased AOR in open surgery with prolonged stay (AOR=12.3, P<0.01)
Vedovati [24]	2014, mRCT	Laparoscopic colorectal cancer surgery	Patients with extended TP (LMWH for 4 weeks, n=112) vs patients w/o extended TP (control, for one week, n=113)	BC was identical in both groups	VTE 0% in extended TP vs 9.7% in control (P=0.001); VTE at 3 months 0.9% vs 9.7% (P=0.005)

Of 11 studies concerning the management of patients undergoing prolonged ATT, seven assessed the safety of preoperative continuation of aspirin during laparoscopic colorectal surgery. In nine studies concerning pharmacological thromboprophylaxis for VTE, patients were generally managed by LMWH. Three studies published in the USA or Italy focused on post-discharge VTE [22-24].

Safety of Laparoscopic Colorectal Cancer Surgery in Patients Undergoing Prolonged ATT (Table 2)

In all 11 studies regarding the management of patients with prolonged ATT, the safety and feasibility of laparoscopic colorectal cancer surgery in ATT-received patients were generally reported.

Seven papers centered on the feasibility of perioperative continuation of aspirin during laparoscopic colorectal resection [2,6-10,15]. One large-scale retrospective cohort study reviewed more than 3,000 patients receiving major gastroenterological malignancy surgery, including 1,445 colorectal cancer resections, and found that the most major risk factor for thromboembolism was the interruption of preoperative antiplatelet treatment [7]. This study also demonstrated that the preoperative continuation of aspirin monotherapy significantly reduced the rate of postoperative thromboembolism, although it was not associated with increased SBL or postoperative BCs. Other six studies, including three retrospective cohort studies and two case-control studies using the propensity score matching method, also showed that preoperative continuation of aspirin is not related to increased rates of BC or SBL in patients with prolonged antiplatelet prescription during laparoscopic colorectal cancer surgery [2,6,8-10,15]. These articles suggest that when performing laparoscopic colorectal cancer surgery in patients with prolonged antiplatelet therapy, preoperative continuation of aspirin is safe and should be considered preferable.

Safety of Pharmacological Thromboprophylaxis for VTE (Table 3)

Among nine articles on pharmacological thromboprophylaxis for VTE, seven were multicenter studies, including four randomized clinical trials and three retrospective cohort studies. Three out of four randomized clinical trials were from Japan [16-18], and it was shown that the occurrence of overall and major BCs under pharmacological prophylaxis during laparoscopic colorectal cancer surgery was relatively higher (1.8-13.1% in overall and 0.7-1.7% in major BCs) compared to those without medical prophylaxis, although its efficacy for VTE is not statistically relevant. Three studies from the United States and Italy demonstrated the relevant rate of post-discharge VTE after laparoscopic colorectal cancer surgery (0.7%). These studies suggest the significance of extended chemical prophylaxis in this patient population [22-24], although no study regarding post-discharge VTE was reported from Asian countries.

Assessment of these studies has suggested a potentially high risk of bleeding in patients receiving pharmacological thromboprophylaxis. On the other hand, the effectiveness of pharmacological thromboprophylaxis after laparoscopic colorectal cancer surgery is not statistically shown, particularly in the Asian population.

Discussion

To our knowledge, the present research is the first systematic review that analyzes the efficacy of ATT on thromboembolism and hemorrhagic complications in laparoscopic colorectal cancer surgery. The current review outlines 20 articles, including a total of 12,832 patients who underwent laparoscopic colorectal cancer surgery, especially in relation to ATT. Most of the studies included in the present research have shown that laparoscopic colorectal cancer surgery can be conducted safely in patients undergoing prolonged ATT, even with preoperative continuation of aspirin. Regarding the pharmacological prevention of VTE, most studies have demonstrated that patients receiving pharmaceutical prophylaxis may have a higher risk of hemorrhagic complications, but their effectiveness against VTE has not been statistically demonstrated, particularly in the Asian patient population.

Since thromboembolism can cause severe sequelae or death, recently updated guidelines for antithrombotic management during non-cardiac surgery state that prevention of thromboembolism is more crucial than suppression of BCs [3,25]. However, advanced laparoscopic surgery, including laparoscopic colorectal resection, showed limited evidence concerning the specific perioperative antithrombotic management guidelines or protocol. 

**Figure 1:**
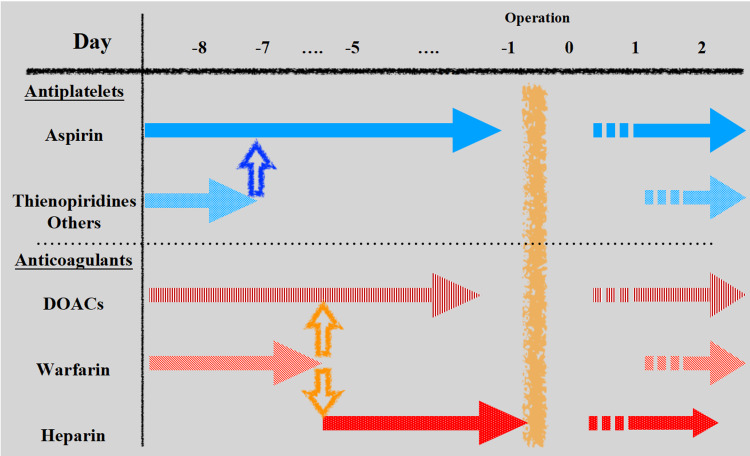
Recommended perioperative management protocol for patients undergoing ATT in the case of gastroenterological surgery. The management generally consists of three ways according to types of ATT: antiplatelet, warfarin, and DOACs. In patients with thromboembolic risks, aspirin monotherapy is continued in patients receiving antiplatelet therapy, and warfarin is substituted by DOAC bridging (preferred) or heparin bridging. In case of DOAC, short-period discontinuation of DOACs (usually 1-2 days) without heparin bridging is generally recommended. Postoperatively, every antithrombotic agent is reinstituted as soon as possible (POD1-2). ATT, antithrombotic therapy; APT, antiplatelet therapy; TE, thromboembolism; ACT, anticoagulation therapy; DOAC, direct-acting oral anticoagulant.

Our facility is a high-volume treatment center for patients with gastroenterological cancer who are using antithrombotic medications. As a result, we utilize a centralized antithrombotic management protocol in patients with prolonged ATT when performing elective surgery, including laparoscopic surgery (Figure 1) [26]. This protocol continues to be updated with reference to some recent guidelines on perioperative antithrombotic management for endoscopic or non-cardiac surgery [3,25]. According to the type of ATT, there are three management options: antiplatelet, warfarin, and DOACs. When patients with thromboembolic risks are given antiplatelet medication, aspirin monotherapy is continued, and warfarin is replaced with DOAC (recommended) or heparin bridging. Short-term withdrawal of DOACs (typically 1-2 days) without heparin bridging is generally indicated in the case of DOACs. Every antithrombotic agent is reintroduced as soon as possible after surgery (postoperative day [POD]1-2).

Regarding the management of patients receiving antiplatelet treatment, several studies, including the POISE-2 study, have demonstrated that continuing antiplatelet therapy during non-cardiac surgery slightly increases the risk of bleeding [27], but most other studies have shown no significant increase in bleeding events [2,7]. In addition, a recently presented large-scale retrospective cohort study has demonstrated that preoperative continuation of aspirin therapy in patients undergoing digestive cancer surgery statistically decreased the incidence of thromboembolism but that it was not related to an increase in hemorrhagic complications [7]. In the present systematic review, seven studies have shown that preoperative continuation of aspirin therapy is not associated with an increase in BC or SBL in patients with prolonged antiplatelet therapy during laparoscopic colorectal cancer surgery [2,6-10,15]. Optimal management of antiplatelet-received patients when performing laparoscopic colorectal cancer surgery is still under discussion, but recommended antithrombotic management, such as continuation of aspirin, should be taken into account.

Concerning VTE prophylaxis during laparoscopic colorectal cancer surgery, most of the studies discussed in the current review have shown that pharmacological thromboprophylaxis potentially increases the risk of BCs, although the effectiveness of VTE prophylaxis has not been shown, especially in the Asian population. VTE is a perioperative, life-threatening complication, and its prevention is crucial. Although Western guidelines recommend pharmacological prophylaxis during non-cardiac surgery [28], racial differences in the occurrence of VTE have been reported between Westerners and Asians [29]. In addition, one systematic review of pharmaceutical prevention of VTE in the Asian patient population has shown that the perioperative risk of VTE is relatively low even in patients with risk factors that are generally considered high risk [29]. A large-scale cohort study conducted in Japan also showed that no clinically relevant VTE was observed in more than 1,000 patients who underwent laparoscopic colorectal cancer surgery [7]. At present, the safety and efficacy of pharmacological VTE prevention during laparoscopic colorectal cancer surgery is still under debate, especially in Asians. It is crucial to build evidence in order to personalize the risks according to race.

Summary and Recommendations for Future Studies

Presently, only a few studies on antithrombotic management during laparoscopic colorectal cancer surgery have been investigated. As society ages, the prevalence of cardiovascular disease is increasing, and there is an urgent need to develop clear protocols or guidelines for perioperative antithrombotic management. The definite guidelines available in the clinical setting need to be established on the basis of well-designed, reliable research. Currently, several promising studies are underway, which are registered in the University Hospital Medical Information Network (UMIN) Clinical Trials Registry [30]. The effectiveness and safety of antithrombotic management during laparoscopic colorectal cancer surgery will be established by the results of such well-designed studies.

## Conclusions

Laparoscopic colorectal cancer surgery in patients receiving prolonged ATT can be safely performed without increasing thromboembolism or hemorrhagic complications. Pharmacological VTE prophylaxis after laparoscopic colorectal cancer surgery is still controversial in terms of efficacy and safety. Establishing a clear guideline or protocol necessitates more research based on credible design research.
